# The internet, teenagers, and sexual health information: a cautionary tale

**DOI:** 10.1186/2045-4015-1-39

**Published:** 2012-09-24

**Authors:** Freya Lund Sonenstein

**Affiliations:** 1 Johns Hopkins Bloomberg School of Public Health, 615 North Wolfe Street, Baltimore, MD, 21205, USA

## Abstract

Although most Israeli teenagers have access to the web and many have used the internet to obtain health information, they do not have access to accurate and complete information about contraceptives on Hebrew language websites. Indeed other evidence suggests that teens do not use the web frequently for health information, they are wary of the information obtained from internet sites, and the search engines that they use may not lead them to the most helpful resources. While the internet has the capacity to provide teenagers with information which can assist them in preventing unintended pregnancy and sexually transmitted infections, interventions that address these challenges need to be developed and rigorously tested.

## 

The author declares that she has no competing interests.

The phenomenal expansion of the availability and use of the web by teenagers, and others, has led to much excitement among health educators and family planning advocates about its promise as a source of accurate information about contraception and other sexuality topics. This new information source could be particularly helpful when access to this sensitive information may be otherwise limited. In the United States, for example, sexuality education in many states’ public schools is prohibited from providing information about contraception because of policies requiring a focus on abstinence until marriage. Religion or culture in many countries can limit access to accurate information about contraception from the traditional purveyors of life skills like family and schools. Might the widely accessible internet fill this void through websites that teens can visit to find out about contraception and how to prevent unintended pregnancy and sexually transmitted infections?

To address this need, the information available on the internet needs to be accurate and complete. The accompanying article by Neumark and colleagues examines the quality of health information about oral contraceptives on Hebrew-Language websites [1]. The findings are worrisome. Reviewing 29 websites the authors find that the information provided was frequently not accurate or complete on a number of items deemed essential according to contraception guidelines developed by a family planning expert. The websites were also assessed for usability and credibility. While all the sites rated highly on usability, credibility varied considerably. Credibility is measured using the Health on the Net (HON) Code of Conduct created by a group of the world’s foremost experts on telemedicine who first met in Geneva in 1995. Unfortunately, the sites providing the most accurate and complete information about oral contraceptive had the lowest ratings on credibility. However, it is somewhat comforting to know that a review of laboratory studies indicates that consumers of websites are more influenced by the way the site is designed and whether it “appears professional” than the indicators developed by experts [2].

What do we know about how teenagers consume the internet and think about information obtained from it? The research about this topic is quite limited. The accompanying article reports that virtually all Israeli 7^th^-12^th^ graders have access to the Internet and more than half had searched the web in the last year for health information [3]. Statistics such as these are used to show that teenagers are big consumers of health information on the internet, but other studies indicate that most of the time that teenagers spend on the internet is not devoted to information seeking. In the US, for example, the most frequent use of the internet by 12–17 year olds is social networking. The next most frequent use is multi-media and entertainment. Compared to older populations teenagers spent the least amount of time visiting health information sites [4]. So although teenagers occasionally seek health information on the internet, they are not doing it frequently. Moreover qualitative data, again collected in the US indicate that teens are mistrustful of the internet as a source of sexual health information [5]. It is likely that the patterns are similar in Israel but only rigorous studies will provide the necessary evidence.

Beyond needing to be motivated to seek information on the internet, teenagers may also be limited by the search engines that they use. If they are using computers in public schools or libraries these engines may have monitors that limit access to certain information. Even without these monitors, consumers seeking information on the internet about contraceptives may not land on the most informative sites. For example, I used Google to search for information about “contraceptives” and “how to prevent pregnancy” from my Johns Hopkins Bloomberg School of Public Health desktop computer and did not find on the first page of the web results, one of the most user friendly sources of information about contraceptives available. Bedsider developed by the National Campaign to Prevent Teenager and Unintended Pregnancy provides accurate information about all methods of contraception as well as information about where to get them and applications to help the consumer chose the right method for herself/himself and to remind the consumer to take their pills or renew their method [6].

All this evidence suggests that in developing the internet as a vehicle for educating teenagers about contraceptive options, much work needs to be done. As the article by Neumark and colleagues indicates, the information needs to be more accurate and complete. But beyond this, sites with a commitment to assisting teenagers in making informed contraceptive choices need to address the search engine problem so that their sites are readily accessible to motivated teenagers. However the biggest challenge may be getting teenagers motivated to become discerning seekers of health information on the internet. At present teenagers do not heavily rely on the web for health information and they appear wary of the sexual health information available there, perhaps suitably so. The internet has the capacity to provide information that can assist teenagers prevent unintended pregnancy and sexually transmitted disease. The field, however, is in its infancy and a systematic and informed effort is needed to develop and test interventions that solve these challenges.

## Competing interest

The author declares that she has no competing interests.

## Author information

Freya Lund Sonenstein Ph.D. is a Professor in the Department of Population, Family and Reproductive Health at the Johns Hopkins Bloomberg School of Public Health. She directs the Center for Adolescent Health, a prevention research center funded by the Centers for Disease Control and Prevention in the U.S. Department of Health and Human Services.
